# SNAP25 differentially contributes to G_i/o_-coupled receptor function at glutamatergic synapses in the nucleus accumbens

**DOI:** 10.3389/fncel.2023.1165261

**Published:** 2023-05-02

**Authors:** Kevin M. Manz, José C. Zepeda, Zack Zurawski, Heidi E. Hamm, Brad A. Grueter

**Affiliations:** ^1^Department of Anesthesiology, Vanderbilt University Medical Center, Nashville, TN, United States; ^2^Department of Pharmacology, Vanderbilt University, Nashville, TN, United States; ^3^Vanderbilt Brain Institute, Vanderbilt University, Nashville, TN, United States; ^4^Vanderbilt Center for Addiction Research, Vanderbilt University, Nashville, TN, United States

**Keywords:** soluble N-ethylmaleimide attachment protein receptor (SNARE), G-protein-coupled receptors (GPCR), SNAP25, nucleus accumbens (NAc), synaptic transmission and plasticity, addiction neural plasticity

## Abstract

The nucleus accumbens (NAc) guides reward-related motivated behavior implicated in pathological behavioral states, including addiction and depression. These behaviors depend on the precise neuromodulatory actions of G_i/o_-coupled G-protein-coupled receptors (GPCRs) at glutamatergic synapses onto medium spiny projection neurons (MSNs). Previous work has shown that discrete classes of G_i/o_-coupled GPCR mobilize Gβγ to inhibit vesicular neurotransmitter release via t-SNARE protein, SNAP25. However, it remains unknown which Gαi/o systems in the NAc utilize Gβγ-SNARE signaling to dampen glutamatergic transmission. Utilizing patch-clamp electrophysiology and pharmacology in a transgenic mouse line with a C-terminal three-residue deletion of SNAP25 (SNAP25Δ3) weaking the Gβγ-SNARE interaction, we surveyed a broad cohort of G_i/o_-coupled GPCRs with robust inhibitory actions at glutamatergic synapses in the NAc. We find that basal presynaptic glutamate release probability is reduced in SNAP25Δ3 mice. While κ opioid, CB1, adenosine A1, group II metabotropic glutamate receptors, and histamine H3 receptors inhibit glutamatergic transmission onto MSNs independent of SNAP25, we report that SNAP25 contributes significantly to the actions of GABA_B_, 5-HT1_B/D_, and μ opioid receptors. These findings demonstrate that presynaptic G_i/o_-coupled GPCRs recruit heterogenous effector mechanisms at glutamatergic synapses in the NAc, with a subset requiring SNA25-dependent Gβγ signaling.

## Introduction

Maladaptive circuit rearrangements in the nucleus accumbens (NAc)-embedded reward network underlie the development of various psychiatric disease states, including addiction (Joffe et al., 2014; Turner et al., 2018) and depression (Russo and Nestler, 2013). The NAc directs reward-related motivational behavior by integrating glutamatergic input from assorted corticolimbic structures (Kalivas, 2009). The strength of these inputs onto medium spiny projection neurons (MSNs) is scaled by plasticity mechanisms at pre- and postsynaptic loci that often require the activity of G_i/o_-coupled G-protein-coupled receptors (GPCRs) (Johnson and Lovinger, 2016). Numerous neuromodulatory systems implicated in addiction-related behavior engage presynaptic G_i/o_-coupled GPCRs at glutamatergic synapses in the NAc, including the GABA_B_ receptor (GABA_B_R) (Li and Slesinger, 2022), adenosine receptors (Ballesteros-Yáñez et al., 2018), cannabinoid receptor type-1 (CB_1_R) (Wiskerke et al., 2008), 5-HT_1B/D_ serotonin receptors (Cao et al., 2013), opioid receptors (Reeves et al., 2022), and the histamine H3 receptor (H_3_R) (Manz et al., 2021a). Although GPCRs hold promise as targets for therapeutic intervention, the precise effector pathways of these receptors at glutamatergic synapses in the NAc remain poorly understood.

Presynaptic G_i/o_-coupled GPCRs decrease neurotransmitter release probability by engaging Gβγ-dependent signaling pathways (Atwood et al., 2014). Mobilization of Gβγ subunits at the presynaptic active zone can decrease terminal Ca^2+^ influx by inhibiting voltage gated Ca^2+^ channels (VGCCs) (Bean, 1989; Ikeda, 1996). Gβγ signaling can also promote inwardly rectifying K^+^ channel (Kir) opening, hyperpolarizing cells below spike threshold and limiting VGCC activity (Kofuji et al., 1995; Hibino et al., 2010). However, recent studies from our lab and others indicate that G_i/o_-coupled GPCR signaling can directly inhibit vesicular release by binding synaptosomal-associated protein of 25 kDa (SNAP25), a member of the soluble N-ethylmaleimide attachment protein receptor (SNARE) complex (Zurawski et al., 2016, 2017; Manz et al., 2019). While mechanisms may be recruited in parallel to exert manifold control over synaptic function, it is unknown which G_i/o_-coupled GPCR systems in the NAc utilize the Gβγ-SNARE interaction.

To address this gap, we employed whole-cell patch-clamp electrophysiology with high-affinity and biased agonist pharmacology in acute brain slices from transgenic mice containing a C-terminus 3-residue deletion of SNAP25 (SNAP25Δ3). Gβγ binds the botulinum toxin type-A cleavage site of SNAP25, disrupting the Ca^2+^-sensitive interaction with vesicular SNARE (v-SNARE) protein, synaptotagmin-1 (Wells et al., 2012). We previously demonstrated that Gβγ has a reduced ability to inhibit the interaction between SNAP25Δ3 and synaptotagmin-1 by approximately 47% compared to WT SNAP25 protein (Zurawski et al., 2019). Therefore, this model would allow us to explore the contributions of the Gβγ-SNARE complex interaction to G_i/o_-coupled GPCR systems in the NAc. We report that basal glutamate release probability is decreased at synapses in the NAc of SNAP25Δ3 mice. Screening G_i/o_-coupled GPCRs canonically confined to presynaptic domains at glutamatergic synapses in the NAc, we show that κ opioid, CB_1_, adenosine A1, and histamine H3 receptors inhibit glutamatergic transmission onto MSNs independent of the Gβγ-SNARE interaction. In contrast, GABA_B_, 5-HT_1B/D_, and μ opioid receptor signaling is Gβγ-SNAP25-dependent, with plasticity elicited by μ opioid receptors completely abolished in SNAP25Δ3 mice. These findings offer insight into the diversity with which G_i/o_-coupled GPCRs modulate glutamatergic transmission in the NAc and yield additional intracellular targets for the treatment of NAc-dependent pathologies.

## Materials and methods

### Animals

Animals were bred and housed at Vanderbilt University Medical Center in accordance to IACUC. Male mice 8–12 weeks of age were used for all electrophysiological experiments. Mice were housed in groups of 2–5/cage on a 12 h light/dark cycle with *ad libitum* access to food and water. SNAP25Δ3 transgenic mice lacking the Gβγ-binding motif at the C-terminus of SNAP25 and WT littermate controls were generated by the Heidi Hamm laboratory (Vanderbilt University).

### Pharmacology

(RS)-Baclofen (BAC), sumatriptan succinate, [D-Ala^2^, NMe-Phe^4^, Gly-ol^5^]-enkephalin (DAMGO), (-)–U50488, WIN 55-212, (1*R*, 4*R*, 5*S*, 6*R*)-4-Amino-2-oxabicyclo [3.1.0] hexane-4,6-dicarboxylic acid (LY379268), histamine dihydrochloride (HA), *N*^6^-Cyclopentyladenosine (CPA), all purchased from Tocris Bioscience. Picrotoxin (PTX) purchased from Sigma Aldrich. Benzyloxy-Cyclopentyladenosine (BnOCPA) was provided by the Hamm Lab.

### Electrophysiology

Whole-cell voltage clamp recordings were performed on MSNs in acute brain slices collected from animals sacrificed under isoflurane anesthesia as described previously (Manz et al., 2021c). Briefly, acute brain slices were prepared from whole brain tissue using a Leica Vibratome in oxygenated (95% O_2_; 5%CO_2_) ice-cold *N*-methyl-d-glucamine (NMDG)-based solution (in mm: 2.5 KCl, 20 HEPES, 1.2 NaH_2_PO_4_, 25 glucose, 93 NMDG, 30 NaHCO_3_, 5.0 sodium ascorbate, 3.0 sodium pyruvate, 10 MgCl_2_, and 0.5 CaCl_2_-2H_2_O). After 8–12 min, slices were transferred into room temperature artificial cerebral spinal fluid (in mM: 119 NaCl, 2.5 KCl, 1.3 MgCl2-6H2O, 2.5 CaCl2-2H2O, 1.0 NaH2PO4-H2O, 26.2 NaHCO3, and 11 glucose; 290–295 mOsm) and MSNs were patched with 4–6 MΩ recording pipettes (pulled with a P-1000 Micropipette Puller; Sutter Instrument) using a Cs + -based intracellular solution (in mM: 120 CsMeSO3, 15 CsCl, 8 NaCl, 10 HEPES, 0.2 EGTA, 10 TEA-Cl, 4.0 Mg-ATP, 0.3 Na-GTP, 0.1 spermine, and 5.0 QX 314 bromide; 290 mOsm). MSNs were distinguished from interneurons in the NAc by morphology (size, shape) as well as biophysical properties (e.g., capacitance, membrane resistance, and AMPAR current decay kinetics). Evoked excitatory postsynaptic currents (EPSCs) were isolated by electrical stimulation through a bipolar electrode in the presence of GABA_A_R antagonist, picrotoxin (PTX: 50 μM). Paired-pulse ratios (PPRs) were obtained by delivering two 0.3 ms duration electrical pulses at 20, 50, 100, 200, and 400 ms interstimulus intervals. Spontaneous EPSCs were collected using a Gap Free protocol in the voltage clamp configuration while cells were held at −70 mV.

### Statistics and data analysis

Data collected from electrophysiology experiments were initially analyzed in Clampfit 10.7. Briefly, peak amplitudes were isolated in Clampfit 10.7 by first adjusting baseline to the average of all traces using cursors placed within the initial 15 ms before stimulation, then cursors were set around EPSCs, being sure to be placed after any stimulation artifact and the minimum peak amplitudes and correlating timepoint were extracted. A MATLAB script was used to average peak amplitudes into averages per minute and converted into percentages. The percentage values were averaged and the mean and SEM were plotted using GraphPad Prism v9.0 and descriptive statistics as well as Student’s *t*-tests were performed using GraphPad Prism. Inclusion criteria for cells were a steady (<20% change) access resistance (RA) of <20 mΩ, and a steady holding current when held at −70 mV. For EPSC analyses, all cells included for analysis had an EPSC average amplitude between 150 and 600 pA.

## Results

### Basal release probability at glutamatergic synapses in the NAc is reduced in SNAP25Δ3 mice

**FIGURE 1:**
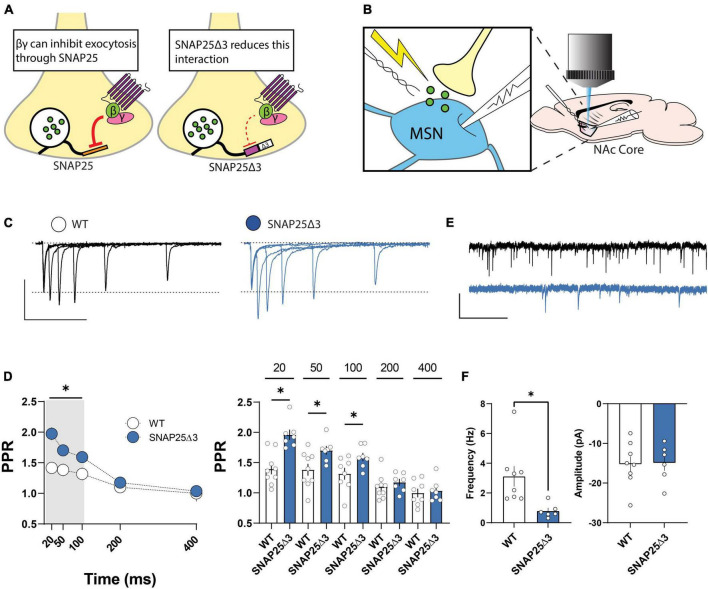
The SNAP25Δ3 mutation results in changes of basal excitatory transmission in the nucleus accumbens. **(A)** SNAP25Δ3 reduces the interaction between SNARE-complex protein SNAP25 and the Gβγ subunits of G_i/o_-coupled GPCRs. **(B)** Experimental setup depicting whole-cell voltage clamp of an MSN in the nucleus accumbens core and electrical stimulation of synaptic terminals to provoke release of presynaptic glutamate. **(C)** Representative traces of EPSCs elicited by stimulation of synaptic terminals at varying interstimulus intervals in WT (black) and SNAP25Δ3 (blue) mice (scale bar: vertical = 100 pA, horizontal = 200 ms). **(D)** Differences in paired-pulse ratios (PPR) shown at 20 ms (WT: mean = 1.396 ± 0.09, SNAP25Δ3: mean = 1.96 ± 0.8; *p* = 0.0005), 50 ms (WT: mean = 1.382 ± 0.10; SNAP25Δ3: mean = 1.70 ± 0.07; *p* = 0.0254), and 100 ms (WT: mean = 1.32 ± 0.10; SNAP25Δ3: mean = 1.59 ± 0.068; *p* = 0.0434), but not 200 ms or 400 ms delays. [WT: *n* = 9 cells; SNAP25Δ3: *n* = 7 cells]. **(E)** Representative traces of spontaneous EPSCs recorded from MSNs from WT (black) and SNAP25Δ3 (blue) mice (scale bar: vertical = 20 pA, horizontal = 1 s). **(F)** Decrease in the frequency of sEPSCs (WT: mean = 3.12 Hz ± 0.69; SNAP25Δ3: mean = 0.80 ± 0.19; *p* = 0.0017) but not amplitude (WT: mean = −15.26 ± 1.98 pA; SNAP25Δ3: mean = −14.94 ± 1.95 pA; *p* = 0.9114) in SNAP25Δ3 mice. [WT: *n* = 8 cells, SNAP25Δ3: *n* = 6 cells]. Error bars indicate SEM. *p < 0.05. Student’s unpaired, two-tailed, *t*-test.

SNAP25Δ3 mice harbor a 3-residue deletion at the C-terminal Gβγ-binding domain of t-SNARE protein, SNAP25, thereby attenuating this interaction under basal conditions (Zurawski et al., 2019; Figure 1A). To determine whether this manipulation elicits adaptations in basal synaptic efficacy, we compared the paired-pulse ratio (PPR) of electrically evoked excitatory postsynaptic currents (EPSCs) at NAc core MSNs in SNAP25Δ3 mice and WT littermates using whole-cell voltage clamp electrophysiology (Figure 1B). PPR was increased at 20, 50, and 100 ms interstimulus intervals (ISIs) at SNAP25Δ3 synapses relative to WT, which disappeared at 200 and 400 ms ISIs (Figures 1C, D). Accordingly, SNAP25Δ3 MSNs exhibited a decrease in the frequency but not amplitude of spontaneous (sEPSCs) relative to WT littermates (Figures 1E, F). These data suggest that SNAP25 contributes to basal synaptic release probability at glutamatergic synapses in the NAc. In contrast, baseline glutamatergic synaptic transmission from cultured hippocampal pyramidal neurons generated from SNAP25Δ3 and WT mice were not different (Alten et al., 2022). Together, these findings demonstrate synaptic specificity of Gβγ-SNARE interactions.

### SNAP25Δ3 disrupts G_i/o_-coupled GPCR-induced reduction in glutamatergic transmission in select GPCR systems in the NAc

**FIGURE 2:**
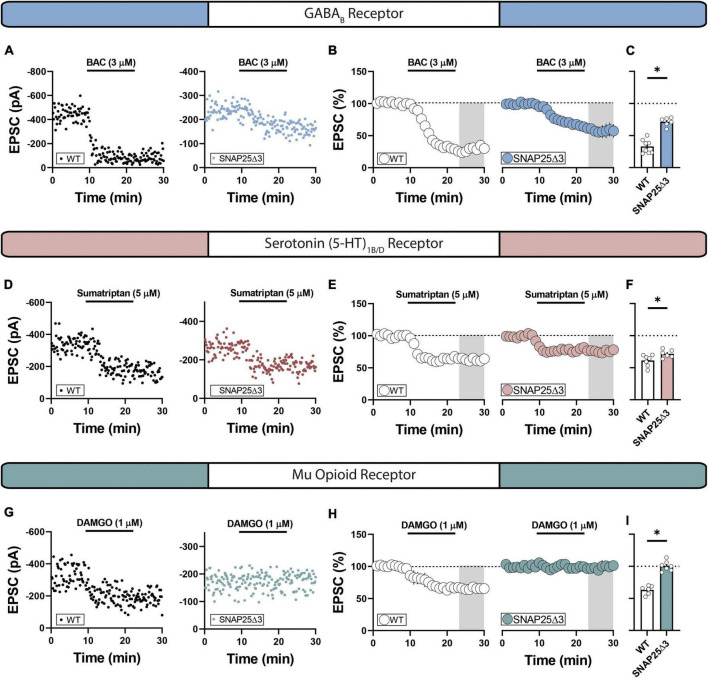
SNAP25Δ3 attenuates G_i/o_-coupled GPCR induced depression of excitatory transmission in select GPCR systems. **(A)** Representative experiments showcasing amplitude peaks of EPSCs following bath application of GABAB receptor agonist baclofen (BAC: final concentration = 3 uM) collected from WT and SNAP25Δ3 mice. **(B)** Percentage changes in EPSC peak amplitude with regards to baseline following BAC application (application time: 10–20 min). **(C)** Summary of average EPSC percentages following BAC application (time = 25–30 min; WT: mean = 33.01 ± 3.77%; SNAP25Δ3: mean = 71.27 ± 2.72%; *p* = < 0.0001). [WT: *n* = 8 cells; SNAP25Δ3: *n* = 6 cells]. **(D)** Representative experiments showcasing amplitude peaks of EPSCs following bath application of 5-HT1B/D receptor agonist sumatriptan (sumatriptan: final concentration = 5 uM) collected from WT and SNAP25Δ3 mice. **(E)** Percentage changes in EPSC peak amplitude with regards to baseline following sumatriptan application (application time: 10–20 min). **(F)** Summary of average EPSC percentages following sumatriptan application (time = 25–30 min; WT: mean 61.16 ± 3.42%; SNAP25Δ3: mean = 71.59 ± 2.52%; *p* = 0.0362). [WT: *n* = 7 cells; SNAP25Δ3: *n* = 6 cells]. **(G)** Representative experiments showcasing amplitude peaks of EPSCs following bath application of Mu-opioid receptor agonist DAMGO (final concentration = 5 uM) collected from WT and SNAP25Δ3 mice. **(H)** Percentage changes in EPSC peak amplitude with regards to baseline following DAMGO application (application time: 10–20 min). **(I)** Summary of average EPSC percentages following DAMGO application (time = 25–30 min; WT: mean 63.15 ± 2.79%; SNAP25Δ3: mean = 100.3 ± 2.90%; *p* = < 0.0001). [WT: *n* = 6 cells; SNAP25Δ3: *n* = 7 cells]. Gray box indicates 5-min bin for comparative analysis. Error bars indicate SEM. *p < 0.05. Student’s unpaired, two-tailed, *t*-test.

We next hypothesized that the Gβγ-SNARE interaction is differentially required for the synaptic effects of select G_i/o_-coupled GPCR systems in the NAc. To interrogate this possibility, we recorded electrically evoked EPSCs in NAc MSNs from SNAP25Δ3 and WT mice before and after pharmacologically activating discrete G_i/o_-coupled GPCRs. We elected to screen GPCR systems previously shown to (1) reduce glutamatergic synaptic efficacy onto MSNs in the NAc (Manz et al., 2019) and (2) typify various addiction-related behavioral phenotypes. Bath-application of the GABA_B_ receptor agonist, baclofen (BAC, 3 μM), partially decreased EPSC amplitude to a significantly greater degree in SNAP25Δ3 mice relative to WT littermates (WT: mean = 33.01 ± 3.77%; SNAP25Δ3: mean = 71.27 ± 2.72%; *p* = < 0.0001) (Figures 2A–C), corroborating prior work showing that SNAP25 contributes to the synaptic effects of GABA_B_ in the NAc (Manz et al., 2019). We next assessed whether the Gβγ-SNAP25 interaction is required for the synaptic effects of the serotonin (5-HT)_1B/D_ heteroreceptor. Indeed, the depression in EPSC amplitude elicited by bath-application of 5-HT_1B/D_ agonist, sumatriptan (5 μM), was modestly but significantly attenuated in SNAP25Δ3 mice (WT: mean 61.16 ± 3.42%; SNAP25Δ3: mean = 71.59 ± 2.52%; *p* = 0.0362) (Figures 2D–F), in agreement with studies where we have shown similar effects in different brain regions and species (Gerachshenko et al., 2005; Zurawski et al., 2019). Interestingly, the μ-opioid receptor agonist, D-Ala^2^, NMe-Phe^4^, Gly-ol^5^]-enkephalin, or DAMGO (1 μM), failed to elicit a depression of EPSCs in SNAP25Δ3 mice (WT: mean 63.15 ± 2.79%; SNAP25Δ3: mean = 100.3 ± 2.90%; *p* = < 0.0001) (Figures 2G–I), suggesting that this interaction is required for the synaptic effects elicited by μ-opioid receptor activity. These data provide electrophysiological evidence that (a) GABA_B_, 5-HT_1B/D_, and μ-opioid receptors in the NAc modulate synaptic function by engaging the Gβγ-SNARE effector system and (b) that these systems are variably disrupted by the SNAP25Δ3 manipulation.

### Synaptic depression elicited by G_i/o_-coupled GPCRs is spared across subtypes in SNAP25Δ3

**FIGURE 3:**
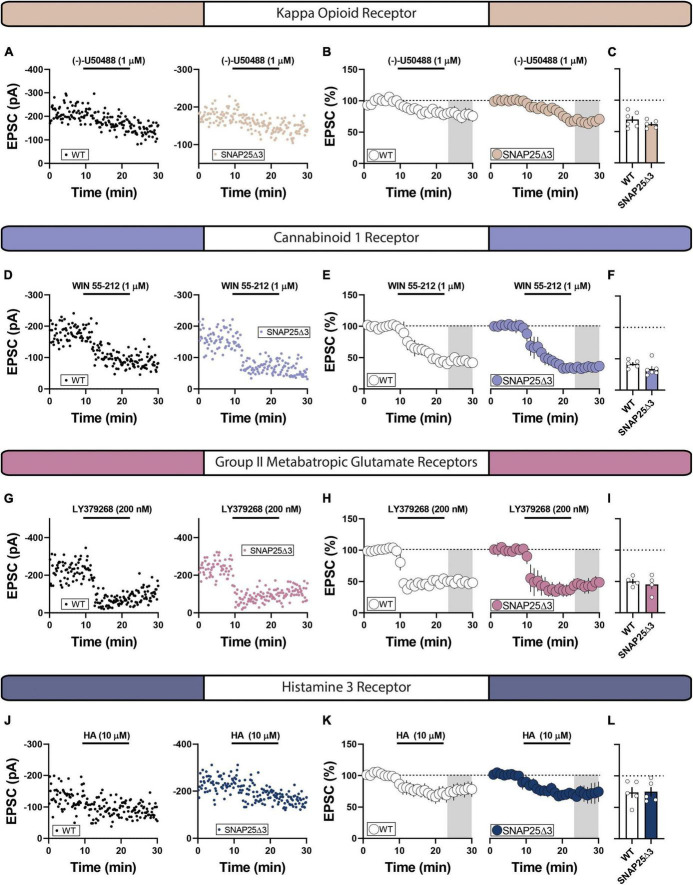
Not all tested G_i/o_-coupled GPCR systems were affected by SNAP25Δ3. **(A)** Representative experiments showcasing amplitude peaks of EPSCs following bath application of κ opioid receptor agonist (-)-U50488 (final concentration = 1 uM) collected from WT and SNAP25Δ3 mice. **(B)** Percentage changes in EPSC peak amplitude with regards to baseline following (-)-U50488 application (application time: 10–20 min). **(C)** Summary of average EPSC percentages following (-)-U50488 application (time = 25–30 min; WT: mean = 65.89 ± 4.80%; SNAP25Δ3: mean = 65.74 ± 4.60%; *p* = 0.9824). [WT: *n* = 5 cells, N = 3 animals; SNAP25Δ3: *n* = 6 cells]. **(D)** Representative experiments showcasing amplitude peaks of EPSCs following bath application of cannabinoid 1 receptor agonist WIN 55–212 (final concentration = 1 uM) collected from WT and SNAP25Δ3 mice. **(E)** Percentage changes in EPSC peak amplitude with regards to baseline following WIN 55–212 application (application time: 10–20 min). **(F)** Summary of average EPSC percentages following WIN 55–212 application (time = 25–30 min; WT: mean 41.67 ± 2.78%; SNAP25Δ3: mean = 33.62 ± 4.57%; *p* = 0.1876). [WT: *n* = 5 cells; SNAP25Δ3: *n* = 6 cells. **(G)** Representative experiments showcasing amplitude peaks of EPSCs following bath application of group II metabotropic glutamate receptor agonist LY379268 (final concentration = 1 uM) collected from WT and SNAP25Δ3 mice. **(H)** Percentage changes in EPSC peak amplitude with regards to baseline following LY379268 application (application time: 10–20 min). **(I)** Summary of average EPSC percentages following LY379268 application (time = 25–30 min; WT: mean 50.08 ± 3.95%; SNAP25Δ3: mean = 45.29 ± 7.56%; *p* = 0.5942). [WT: *n* = 4 cells; SNAP25Δ3: *n* = 4 cells]. **(J)** Representative experiments showcasing amplitude peaks of EPSCs following bath application histamine 3 receptor agonist histamine (HA final concentration = 10 uM) collected from WT and SNAP25Δ3 mice. **(K)** Percentage changes in EPSC peak amplitude with regards to baseline following HA application (application time: 10–20 min). **(L)** Summary of average EPSC percentages following HA application (time = 25–30 min; (WT: mean = 73.61 ± 8.36%; SNAP25Δ3: mean = 74.69 ± 7.38%; *p* = 0.9252). [WT: *n* = 5 cells; SNAP25Δ3: *n* = 5 cells]. Gray box indicates 5-min bin for comparative analysis. Error bars indicate SEM. Student’s unpaired, two-tailed, *t*-test.

To survey a broader cohort of presynaptic GPCR systems, we next screened CB_1_, κ opioid, histamine H3, and group II metabotropic glutamate receptors (mGluRs). Bath-application of the κ opioid receptor agonist, (-)-U50488 (1 μM), induced a similar depression of EPSCs in NAc core slices from SNAP25Δ3 and WT mice (WT: mean = 65.89 ± 4.80%; SNAP25Δ3: mean = 65.74 ± 4.60%; *p* = 0.9824) (Figures 3A–C). Similarly, CB_1/2_ receptor agonist, WIN 55–212 (1 μM), and group II metabotropic glutamate receptor (mGluR) agonist, LY379268 (200 nM), induced robust depression of EPSCs (WT: mean 41.67 ± 2.78%; WIN SNAP25Δ3: mean = 33.62 ± 4.57%; *p* = 0.1876) (Figures 3D–F) that did not differ across genotypes (WT: mean 50.08 ± 3.95%; LY SNAP25Δ3: mean = 45.29 ± 7.56%; *p* = 0.5942) (Figures 3G–I). Although histamine (HA) is not specific for the histamine 3 receptor, we previously showed that HA elicits long-term depression (LTD) of glutamatergic transmission at MSN synapses in the NAc in a histamine H_3_ receptor-dependent manner expressed at presynaptic loci (Manz et al., 2021a,b). Indeed, histamine (10 μM) caused a similar depression of EPSCs in slices from both SNAP25Δ3 and WT mice (WT: mean = 73.61 ± 8.36%; SNAP25Δ3: mean = 74.69 ± 7.38%; *p* = 0.9252) (Figures 3J–L). Taken together, these data suggest GPCR specificity in recruiting the SNAP25 C-terminus as a mechanism to decrease synaptic release probability in the NAc.

### Adenosine A1 receptor mediated depression of glutamatergic transmission does not proceed via SNAP25 in the NAc

**FIGURE 4:**
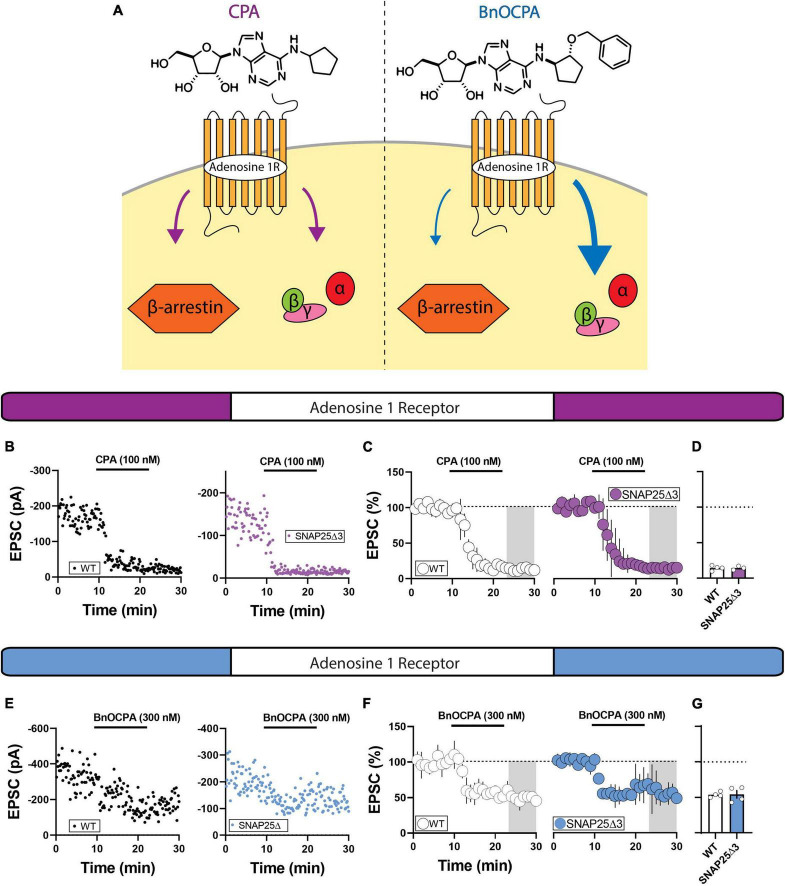
Using a biased agonist of the adenosine A1 receptor to preferentially engage G-protein signaling. **(A)** The biased adenosine A1 receptor BnOCPA preferentially stimulates G-protein signaling. **(B)** Representative experiments showcasing amplitude peaks of EPSCs following bath application adenosine 1 receptor agonist CPA (CPA final concentration = 101 nM) collected from WT and SNAP25Δ3 mice. **(C)** Percentage changes in EPSC peak amplitude with regards to baseline following CPA application (application time: 10–20 min). **(D)** Summary of average EPSC percentages following application of the full adenosine A1 receptor agonist CPA (WT: mean = 14.26 ± 1.72%; SNAP25Δ3: mean = 14.23 ± 1.62%; *p* = 0.9907) [WT: *n* = 4 cells, N = 3 animals; SNAP25Δ3: *n* = 3 cells, N = 3 animals]. **(E)** Representative experiments showcasing amplitude peaks of EPSCs following bath application adenosine 1 receptor agonist CPA (CPA final concentration = 101 nM) collected from WT and SNAP25Δ3 mice. **(F)** Percentage changes in EPSC peak amplitude with regards to baseline following CPA application (application time: 10–20 min). **(G)** Summary of average EPSC percentages following application of the biased adenosine A1 receptor agonist BnOCPA (WT: mean = 51.34 ± 2.30%; SNAP25Δ3: mean = 49.95 ± 7.02%; *p* = 0.8569). [WT: *n* = 4 cells; SNAP25Δ3: *n* = 4 cells). Gray box indicates 5-min bin for comparative analysis. Error bars indicate SEM. Student’s unpaired, two-tailed, *t*-test.

An inherent limitation of gross pharmacological inspection of GPCRs at synaptic loci is their participation in parallel effector pathways. To begin to address this issue, we leveraged the biased adenosine A1 receptor agonist, BnOCPA, to augment G-protein-directed effector signaling (Figure 4A; Wall et al., 2022). Congruent with prior studies, we observed a robust depression of EPSCs with application of adenosine A1 receptor agonist, CPA, in WT animals (Fritz et al., 2021) that was similar to the depression observed in SNAP25Δ3 mice (Figures 4B–D; 100 nM) (WT: mean = 14.26 ± 1.72%; SNAP25Δ3: mean = 14.23 ± 1.62%; *p* = 0.9907). When we applied the biased adenosine A1 receptor agonist, BnOCPA (300 nM), we observed a depression in EPSC amplitude that was indistinguishable between WT and SNAP25Δ3 mice (Figures 4E–G) WT: mean = 51.34 ± 2.30%; SNAP25Δ3: mean = 49.95 ± 7.02%; *p* = 0.8569). Lack of a difference in synaptic depression in response to adenosine A1 receptor full and biased agonists suggests that the adenosine A1 receptor system does not recruit the Gβγ-SNARE to induce the depression of glutamatergic synapses.

## Discussion

The NAc receives robust glutamatergic input from limbic and paralimbic centers guiding motivational decision-making. Experience shifts NAc circuit activity by scaling the strength of these inputs through GPCR-dependent plasticity mechanisms, including G_i/o_-coupled GPCRs implicated in addiction and depression (Lüscher and Malenka, 2011; Grueter et al., 2012). G_i/o_-coupled GPCRs, including those mediating endogenous opioid, cannabinoid, and GABA signaling, canonically inhibit presynaptic release probability through Gαi/o and Gβγ-dependent pathways involving VGCCs, Kirs, and others. However, G_i/o_-coupled GPCRs in the NAc, hippocampus, and BNST have recently been shown to inhibit vesicular neurotransmitter release through a Gβγ-dependent assembly with SNARE proteins such as SNAP25 (Zurawski et al., 2019). Here, we obtained a rigorous functional inventory of key G_i/o_-coupled GPCRs in the NAc that reduce glutamatergic synaptic efficacy through the Gβγ-SNARE interaction. We report that select GPCR systems, each with temporally distinct plasticity patterns, engage SNAP25-dependent effector systems at glutamatergic synapses onto MSNs in the NAc.

First, we aimed to determine the consequences of the SNAP25Δ3 mutation on glutamatergic transmission in the nucleus accumbens. We found that the SNAP25Δ3 mice have a enhanced PPR and reduced sEPSC frequency when compared to WT animals (Figures 1C–F), which are both indicative of reduced vesicular release probability. This result is surprising in that the Gβγ-SNARE interaction is known to decrease vesicular release probability, and this interaction is compromised in the SNAP25Δ3 mice. One possibility is that because the SNAP25Δ3 mutation is present developmentally, compensatory mechanisms are reducing the probability of vesicle fusion to compensate for the loss of inhibition via the Gβγ-SNARE interaction. The basal changes in glutamatergic transmission are an important caveat in this study, however, further studies are needed to elucidate which specific mechanisms are driving the decrease in glutamatergic vesicular release probability at these synapses. Our data suggest that GABA_B_, 5-HT1_B/D_, and μ opioid heteroreceptors differentially engage SNAP25. These findings share electrophysiological similarities in that each GPCR system has been shown in early studies to reduce the frequency of action potential-independent quantal synaptic events recorded as miniature EPSCs (mEPSCs) in MSNs of the NAc (Muramatsu et al., 1998; Ma et al., 2012; Manz et al., 2019). Although these data corroborate a presynaptic localization of function, a VGCC-independent control mechanism has been invoked to support an effect observed with mEPSCs. Studies from our group and others suggest that GABA_B_, a G_i/o_-coupled GPCR well-known for its Gβγ-VGCC interaction (Hamid et al., 2014; Church et al., 2022), inhibits glutamatergic transmission in the NAc independent of N- and P/Q-type VGCCs (Manz et al., 2019; Zurawski et al., 2019). Similar to data obtained here, we report that GABA_B_ instead engages a SNAP25-dependent effect on vesicular release. It’s of interest that in hippocampal CA1 neurons, GABA_B_ inhibits glutamate release independent of Gβγ-SNARE interaction, indicating tissue-specific effects (Alten et al., 2022). To our surprise, the μ opioid receptor-induced decrease in glutamatergic transmission was completely abolished in SNAP25Δ3 mice, highlighting a GPCR system entirely contingent on the Gβγ-SNARE interaction.

Despite a pharmacological screen revealing SNAP25-dependent GPCR systems at glutamatergic synapses in the NAc, a broader group of GPCRs were functionally indistinguishable in SNAP25Δ3 and WT controls, including κ opioid, CB1, adenosine A1, histamine H3, and group II metabotropic glutamate receptors. It is tempting to suggest that these data capture the behavior of the selected GPCRs within the NAc. However, glutamatergic transmission was evoked using local field stimulation of heterogenous afferents, each with distinct GPCR profiles, receptor densities, and presynaptic microenvironments. The adenosine A1 receptor, for example, may recruit input-specific effector systems that are inadequately sampled in our experimental configuration (Fritz et al., 2021). Further studies are needed to delineate whether each GPCR system obeys input- or cell-type-specific signaling heuristics in the NAc.

We hypothesized that by employing the biased agonist BnOCPA, which preferentially engages G-protein signaling as opposed to β-arrestin signaling, we would amplify the Gβγ-signaling component provoked by adenosine A1 receptor agonism. Previous studies have shown that in addition to receptor internalization, β-arrestin can promote diverse signaling cascades that can recruit synaptic plasticity (Hall et al., 1999; Lefkowitz and Shenoy, 2005; Dhami and Ferguson, 2006), we therefore aimed to determine whether Gβγ-SNARE signaling may be engaged by A1 receptor but perhaps occluded by simultaneous β-arrestin signaling by using the G-protein signaling biased A1R agonist BnOCPA. We did not observe differences in EPSC amplitude between WT and SNAP25Δ3 when we applied BnOCPA (Figure 4G), providing us with greater confidence that the Gβγ-SNARE signaling interaction is not necessary for adenosine 1 receptor depression of excitatory synaptic transmission in the NAc. G-protein biased agonists are not available for all of the receptors assessed but are a future avenue for exploring whether some of these receptor systems may still engage the Gβγ-SNARE motif for their synaptic depression.

Gβγ subunits have been shown to have different affinities for SNAP25 (Smrcka, 2008; Yim et al., 2021). An intriguing question is whether the Gβγ-SNARE signaling interaction is directed by GPCRs coupled to Gβγ complexes comprised of specific subunits. Indeed, we have evidence of this GPCR specificity for certain Gβγ subunits that then impact which Gβγ subunits bind SNARE (Yim et al., 2019, 2021). Thus, only those Gβγ subunits with sufficient affinity for SNARE can elicit a presynaptic effect on release probability. Although beyond the scope of this study, careful examination of the Gβγ subunits bound to GPCRs surveyed here would offer additional mechanistic insight into the specificity of our findings. An alternative hypothesis is that SNAP25-dependent GPCR signaling is spatially restricted to those GPCR classes positioned at or near the active zone. As an extension of this hypothesis is the possibility of scaffolding factors which are important for determining localization to the active zone. Nanodomain resolution would be required to resolve whether differential localization of these GPCRs predicts the efficacy with which the Gβγ-SNARE interaction can influence synaptic transmission. An important limitation of our studies is the inability to isolate the Gβγ signaling component by these GPCRs from simultaneously activated pathways such as Gα and β-arrestin (DeWire et al., 2007). Therefore, it is possible that some of the GPCR systems we examined may still engage the Gβγ-SNARE interaction to exert their depression, but that the contributions of this interaction may have been occluded by pathways activated in parallel.

Several of these GPCR systems have been the targets of therapeutics for addiction, though activation of these receptors has been shown to have different behavioral outcomes in addiction models. For example, the GABA_B_ receptor agonist baclofen has been found to reduce drug-craving, drug-seeking, and drug relapse in both humans and rodent models (Hotsenpiller and Wolf, 2003; Kahn et al., 2009), but cannabinoid 1 receptor agonists are able to increase cocaine and heroin relapse (De Vries et al., 2001; Parolaro et al., 2007). Conversely, blocking cannabinoid 1 receptors in the NAc inhibits the reinstatement of drug seeking of heroin (Alvarez-Jaimes et al., 2008), morphine (Yuan et al., 2013), and cocaine (Xi et al., 2006). A deeper understanding of the mechanistic function by these GPCRs may provide important insight for targeting of downstream effector systems for treating addiction. We have successfully identified GPCR systems which, in naïve mice, mediate depression of EPSCs through the SNAP25-Gβγ interaction. Our results provide impetus for the exploration of this effect in additional GPCR systems, inhibitory transmission, brain regions, cell-types, and how these systems are altered in addiction.

## Data availability statement

The raw data supporting the conclusions of this article will be made available by the authors, without undue reservation.

## Ethics statement

The animal study was reviewed and approved by the Vanderbilt University IACUC through Office of Animal Welfare Assurance.

## Author contributions

KM, JZ, HH, and BG designed the experiments and interpreted the results. KM and JZ performed the experiments. KM, JZ, and BG wrote the manuscript. All authors contributed to the article and approved the submitted version.
